# Redox requirements for ubiquitin-like urmylation of Ahp1, a 2-Cys peroxiredoxin from yeast

**DOI:** 10.1016/j.redox.2020.101438

**Published:** 2020-01-22

**Authors:** Cindy Brachmann, Lars Kaduhr, André Jüdes, Keerthiraju Ethiraju Ravichandran, James D. West, Sebastian Glatt, Raffael Schaffrath

**Affiliations:** a Universität Kassel, Institut für Biologie, Fachgebiet Mikrobiologie, Heinrich-Plett-Str. 40, 34132, Kassel, Germany; b Max Planck Research Group at the Malopolska Centre of Biotechnology, Jagiellonian University, 30-387, Krakow, Poland; c Postgraduate School of Molecular Medicine, 02-091, Warsaw, Poland; d Biochemistry & Molecular Biology Program, Departments of Biology and Chemistry, The College of Wooster, Wooster, OH, USA

**Keywords:** *Saccharomyces cerevisiae*, 2-Cys peroxiredoxin, Ahp1, Redox-active thiols, Dimer interface, Thioredoxin system, Ubiquitin-related modifier Urm1, Protein urmylation

## Abstract

The yeast peroxiredoxin Ahp1, like related anti-oxidant enzymes in other species, undergoes urmylation, a lysine-directed conjugation to ubiquitin-like modifier Urm1. Ahp1 assembles into a homodimer that detoxifies peroxides via forming intersubunit disulfides between peroxidatic and resolving cysteines that are subsequently reduced by the thioredoxin system. Although urmylation coincides with oxidative stress, it is unclear how this modification happens on a molecular level and whether it affects peroxiredoxin activity. Here, we report that thioredoxin mutants decrease Ahp1 urmylation in yeast and each subunit of the oxidized Ahp1 dimer is modified by Urm1 suggesting coupling of urmylation to dimerization. Consistently, Ahp1 mutants unable to form dimers, fail to be urmylated as do mutants that lack the peroxidatic cysteine. Moreover, Ahp1 urmylation involves at least two lysine residues close to the catalytic cysteines and can be prevented in yeast cells exposed to high organic peroxide concentrations. Our results elucidate redox requirements and molecular determinants critical for Ahp1 urmylation, thus providing insights into a potential link between oxidant defense and Urm1 utilization in cells.

## Graphical abstract

**Figure:**
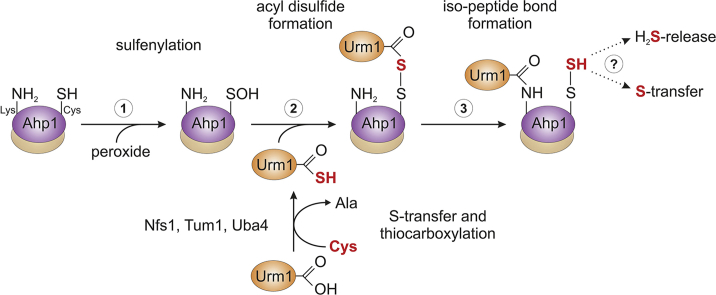

## 1 Introduction

Glutathione peroxidases and peroxiredoxins are highly conserved thiol-dependent proteins, which detoxify various reactive oxygen species (ROS) and thus are critical to maintain cellular redox homeostasis [[1], [2], [3]]. Accordingly, organisms with defects in these anti-oxidant enzymes exhibit premature aging, impaired growth and compromised fitness [[4], [5], [6]]. The yeast *Saccharomyces cerevisiae* expresses several thiol-dependent oxidoreductases, including glutathione peroxidases (Gpx1-Gpx3) and various peroxiredoxins (Ahp1, Dot5, Prx1, Tsa1, Tsa2) [7]. Their number together with differential localization and expression patterns suggests functional plasticity in protection against various ROS including H_2_O_2_ or organic peroxides [8]. In support of this notion, Gpx3 and Tsa1 display broad ROS substrate specificities, while Ahp1 preferentially reduces organic peroxides (e.g. *tert*-butyl hydroperoxide [t-BOOH]) [[9], [10], [11], [12], [13]].

**Fig. 1:**
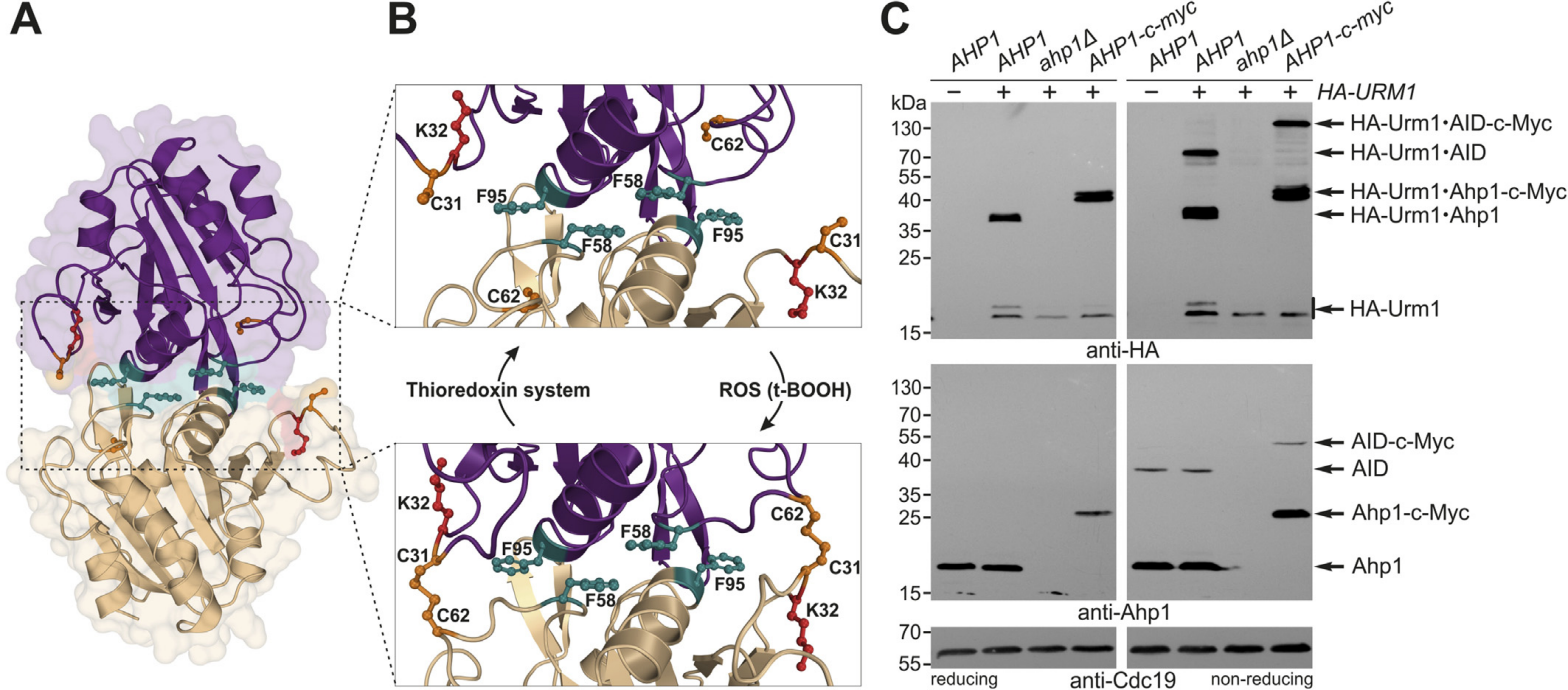
Ahp1 structure, redox states and *in vivo* urmylation. (**A**) Overview of the Ahp1 homodimer (PDB #4DSR, 4DSQ) composed of two subunits (magenta & beige). Highlighted are residues critical for dimerization (F58 & F95: teal), peroxidase activity (C31 & C62: orange) or known urmylation (K32: red). (**B**) The enlargement (top panel) shows the redox-active centers formed between each subunit by resolving (C31) and peroxidatic (C62) thiols. Upon oxidation by ROS (t-BOOH), they become disulfide-bridged (bottom panel) and can be reduced by the thioredoxin system (see Fig. 2A). (**C**) Formation of HA-Urm1•Ahp1 conjugates *in vivo*. Shown are EMSAs under reducing (left panels) and non-reducing (right panels) conditions on protein extracts from indicated strains expressing *HA-URM1* (+) or not (−). NEM-stabilized urmylation was studied by anti-HA blots (top panels) diagnostic for free HA-Urm1 (~17 kDa) and urmylated forms of Ahp1 (~36 kDa) or Ahp1 intersubunit disulfide (AID ~72 kDa) as well as Urm1-modified c-Myc tagged Ahp1 (~43 kDa) or AID (~90 kDa) forms. anti-Ahp1 Western blots (middle panels) detect unmodified Ahp1 (~19 kDa) and AID (~38 kDa) or c-Myc tagged Ahp1 (~27 kDa) and AID (~54 kDa). Protein loading control used anti-Cdc19 blots (bottom panels). (For interpretation of the references to colour in this figure legend, the reader is referred to the Web version of this article.)

Ahp1 is a typical 2-Cys peroxiredoxin, which forms constitutive homodimers by employing phenylalanine residues (Phe-58, Phe-95) that build a hydrophobic dimerization interface (Fig. 1A and B) for precise positioning of the two subunits [14,15]. Each Ahp1 subunit carries resolving (Cys-31: C_R_) and peroxidatic (Cys-62: C_P_) thiols critical for anti-oxidant function [11,12,15,16] (Fig. 1B). During t-BOOH detoxification, the C_P_ thiols become sulfenylated (-SOH), form Ahp1 intersubunit disulfides with the C_R_ thiols and are reduced for a new peroxidatic cycle by thioredoxin (Fig. 1B). Ahp1 also undergoes post-translational modifications, including S-glutathionylation [14] and urmylation. The latter involves lysine-directed conjugation of ubiquitin-related modifier Urm1 [[17], [18], [19], [20], [21], [22]].

**Fig. 2:**
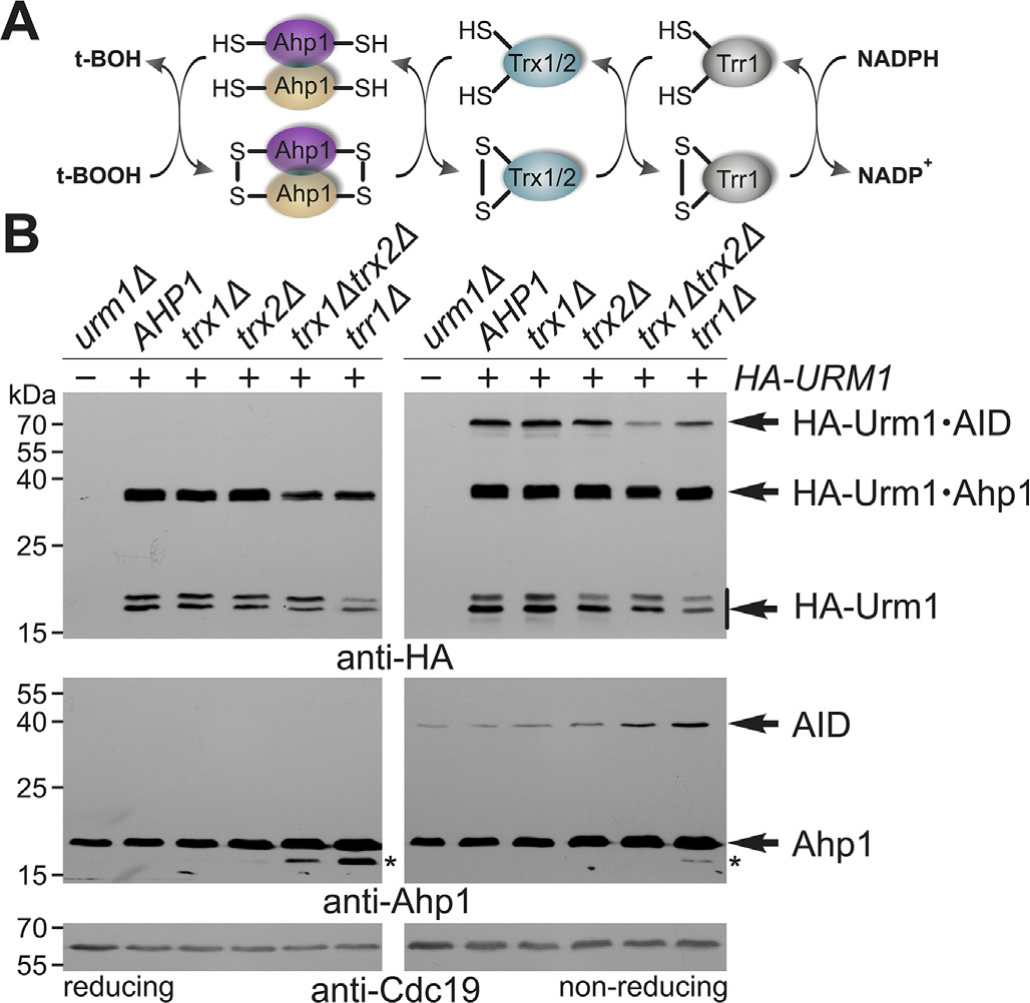
Thioredoxin function supports Ahp1 urmylation. (**A**) Ahp1 oxidation by ROS (t-BOOH) and reduction by the thioredoxin system, i.e. NADPH-dependent thioredoxin reductase (Trr1) and thioredoxins (Trx1; Trx2). (**B**) EMSAs under reducing (left panels) and non-reducing (right panels) conditions with protein extracts of indicated strains expressing *HA-URM1* (+) or not (−). Urmylation was studied by anti-HA (top panels) diagnostic for free HA-Urm1 and urmylated forms of Ahp1 (~36 kDa) or Ahp1 intersubunit disulfides (AID ~72 kDa). anti-Ahp1 blots (middle panels) detect unmodified Ahp1 (~19 kDa) and AIDs (~38 kDa). anti-Cdc19 (bottom panels) served as loading control. Asterisks denote faster migrating Ahp1 forms.

Urm1 has two distinct cellular roles, namely as a post-translational protein modifier and as a sulfur donor for a thiolase (Ncs2-Ncs6), which in concert with the Elongator complex (Elp1-Elp6) adds thiomodifications to wobble uridines in tRNA anticodons [[23], [24], [25], [26], [27], [28]]. Importantly, tRNA thiolation and protein urmylation require sulfur activation and transfer onto the C-terminus of Urm1 by Uba4, an E1-like activator protein that produces thiocarboxylated Urm1 (Urm1-COSH) [17,19,21,29]. Thus, the two Urm1 functions are chemically linked by sulfur transfer and potentially coupled to oxidative stress [19,21,22,30,31].

Consistently, intracellular ROS and other thiol-reactive agents (e.g. *N*-ethylmaleimide [NEM], diamide) trigger urmylation in eukaryotes. Among others, Urm1 targets from yeast, fungi, flies or human cells include oxidative stress response factors like hemopexin, carbonyl reductase and 2-Cys peroxiredoxins (e.g. Prx5, Ahp1) [[18], [19], [20], [21], [22],^32^,^33^]. Urm1•Uba4 modifier systems are exchangeable from yeast to plant and human cells, and, when expressed in yeast, human URM1 conjugates with Ahp1, indicating that protein urmylation is a process conserved among eukaryotes [19,20,34]. Furthermore, the identification of prokaryotic Urm1 or Uba4 counterparts implies that Urm1-like systems and protein urmylation are found throughout evolution [[35], [36], [37]].

Although its biological function remains elusive, it is accepted that urmylation does not act as an ubiquitin-like tag for protein degradation in eukaryotes [18,20,38]. An Urm1 acceptor site identified in Ahp1 (Lys-32) [21] maps next to the C_R_ (Cys-31) (Fig. 1B), suggesting urmylation may interfere with the enzyme's peroxidase activity [15]. Even though urmylation coincides with oxidative stress, it is unknown whether it affects the activity of peroxiredoxins in yeast, insects or human cells. Therefore, we examined urmylation in further detail in *S. cerevisiae* and found that the ability of Ahp1 to form dimers with intact redox-active centers is intimately linked to Urm1 acceptor activity. In addition, Urm1 target site analysis reveals two residues (Lys-32, Lys-156) in proximity to the redox-active center, which when mutated in Ahp1 drastically decrease urmylation without compromising protection against t-BOOH. Our data indicate that oxidative stress and anti-oxidant activity of Ahp1 are required for urmylation.

## 2 Results

### 2.1 Urm1•Ahp1 conjugation based on electrophoretic mobility shift assays (EMSA)

In the presence of isopeptidase inhibitor NEM, Ahp1 is among the most prominent Urm1 targets in *S. cerevisiae* [18,19,21]. Previous studies used β-mercaptoethanol (β-ME) or dithiothreitol (DTT), reducing agents that impede analysis of Urm1 conjugation to Ahp1 intersubunit disulfides [[18], [19], [20], [21]]. Since these disulfides form during the Ahp1 catalytic cycle [15,39,40] (Fig. 1B), we compared urmylation under reducing conditions (standard SDS-PAGE with β-ME in sample buffer) and non-reducing ones (without β-ME) in yeast cells expressing HA-tagged Urm1 (Fig. 1C). In the presence of NEM and β-ME, anti-HA EMSA distinguished free HA-Urm1 (~17 kDa) from a major HA-Urm1 conjugate (~36 kDa) that is absent from the *ahp1Δ* null-mutant and up-shifted (~43 kDa) upon c-Myc tagging in *AHP1-c-myc* cells (Fig. 1C). Thus, the ~36 kDa band represents an Urm1 modified Ahp1 subunit most likely originating under reducing SDS-PAGE conditions from an urmylated dimer.

Accordingly, under non-reducing conditions, we detected a slower migrating HA-Urm1•Ahp1 conjugate roughly double in size (~72 kDa), which is absent from *ahp1Δ* cells and up-shifted (~90 kDa) upon c-Myc tagging (Fig. 1C). This conjugate corresponds to an oxidized dimer with both Ahp1 subunits urmylated and interlinked by disulfides that are sensitive to reduction by β-ME (Fig. 1C). Oxidized Ahp1 dimers detected by anti-Ahp1 EMSA lack urmylation (Fig. 1C), suggesting that attachment of HA-Urm1 (~17 kDa) blocks Ahp1 (~19 kDa) recognition and immune detection by the antiserum [39]. As unmodified Ahp1 remains detectable under our experimental conditions there seems to be an equilibrium of free and urmylated Ahp1 *in vivo*. In support of this notion, independent studies with TAP-tagged Urm1 show differential Ahp1 conjugates including Ahp1 disulfides, which under non-reducing conditions, have one TAP-Urm1 copy attached to each subunit (Suppl. Fig. S1). The occurrence of detectable disulfide-linked dimers modified by Urm1 implies that urmylation does not affect the redox-active thiols required for disulfide formation. Collectively, our data uncover disulfide bridged Ahp1•Urm1 conjugates that coexist with a pool of the Ahp1 peroxiredoxin that is not modified by Urm1.

### 2.2 Defects in the thioredoxin system affect Urm1 conjugation to Ahp1

Urm1 is attached to oxidized Ahp1 intermediates (Fig. 1C) that may be subject to reduction by the cytosolic thioredoxin system (Trx1, Trx2, Trr1) (Fig. 2A) [7]. Therefore, we examined urmylation in yeast strains lacking a functional thioredoxin system. As judged from anti-Ahp1 and anti-HA EMSAs, Ahp1 urmylation and expression levels did not differ in mutants lacking thioredoxin Trx1 (*trx1Δ*) or Trx2 (*trx2Δ*) (Fig. 2B). However, loss of both reducing enzymes (*trx1Δtrx2Δ*) or thioredoxin reductase (*trr1Δ*) lowered urmylation mildly and significantly increased formation of non-modified intersubunit disulfides (Fig. 2B). Accordingly, urmylated disulfides decreased with *trx1Δtrx2Δ* or *trr1Δ* mutants (Fig. 2B). Thus, our data indicate proper recycling of Ahp1 by the thioredoxin system impacts Urm1 conjugation, in particular the formation of urmylated Ahp1 intersubunit disulfides (Fig. 2B).

### 2.3 Dose-dependent suppression of Ahp1 urmylation by t-BOOH

**Fig. 3:**
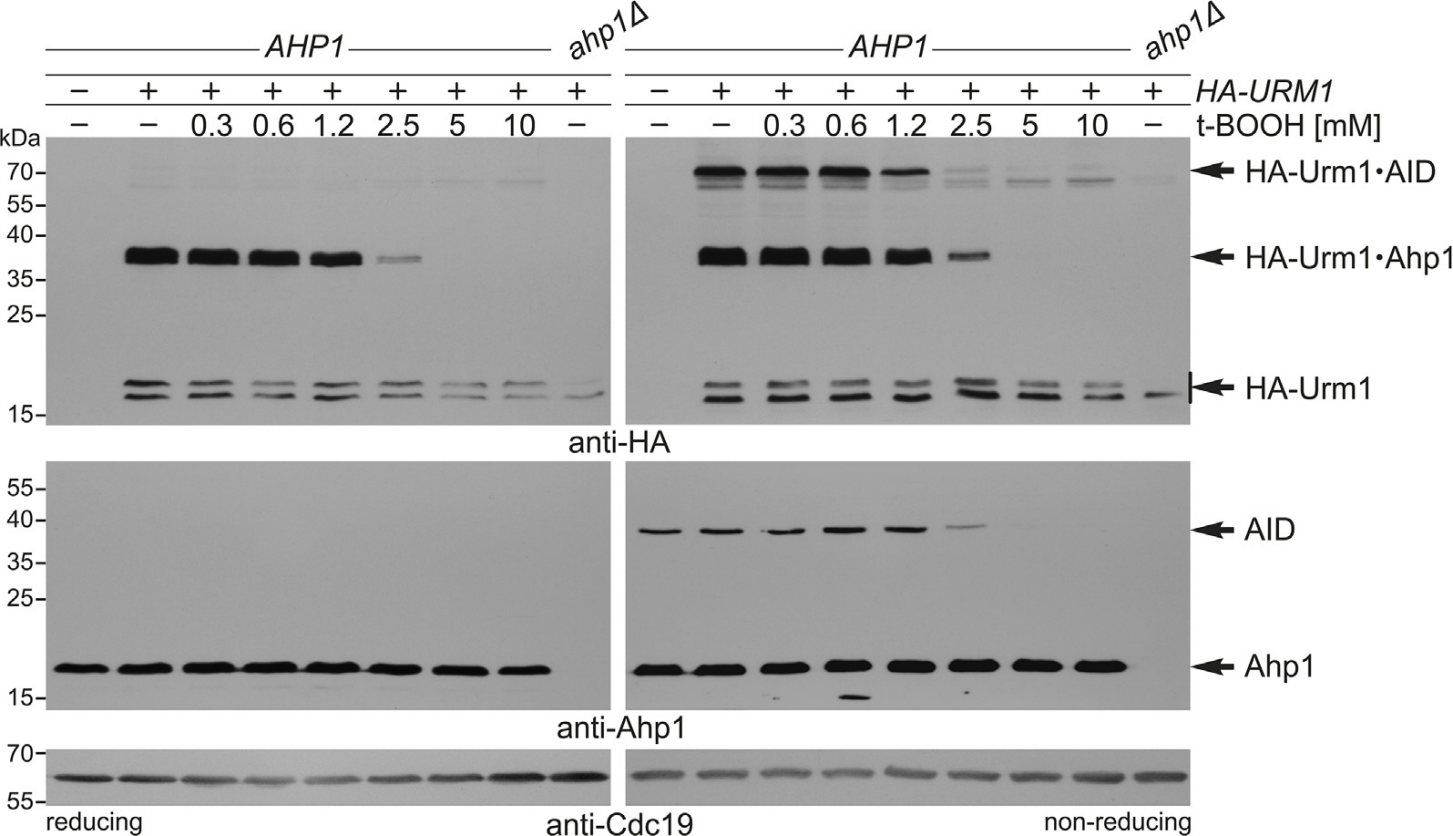
Suppression of Ahp1 urmylation and disulfide formation by t-BOOH *in vivo*. Shown are EMSAs under reducing (left panels) and non-reducing (right panels) conditions from strains treated with t-BOOH as indicated and expressing *HA-URM1* (+) or not (−). NEM-stabilized HA-Urm1 conjugation was studied by anti-HA blots (top panels) diagnostic for free HA-Urm1 and urmylated forms of Ahp1 (~36 kDa) and Ahp1 intersubunit disulfides (AID ~72 kDa). anti-Ahp1 blots (middle panels) detect unmodified Ahp1 (~19 kDa) and AID (~38 kDa). anti-Cdc19 (bottom panels) served as internal standard.

**Fig. 4:**
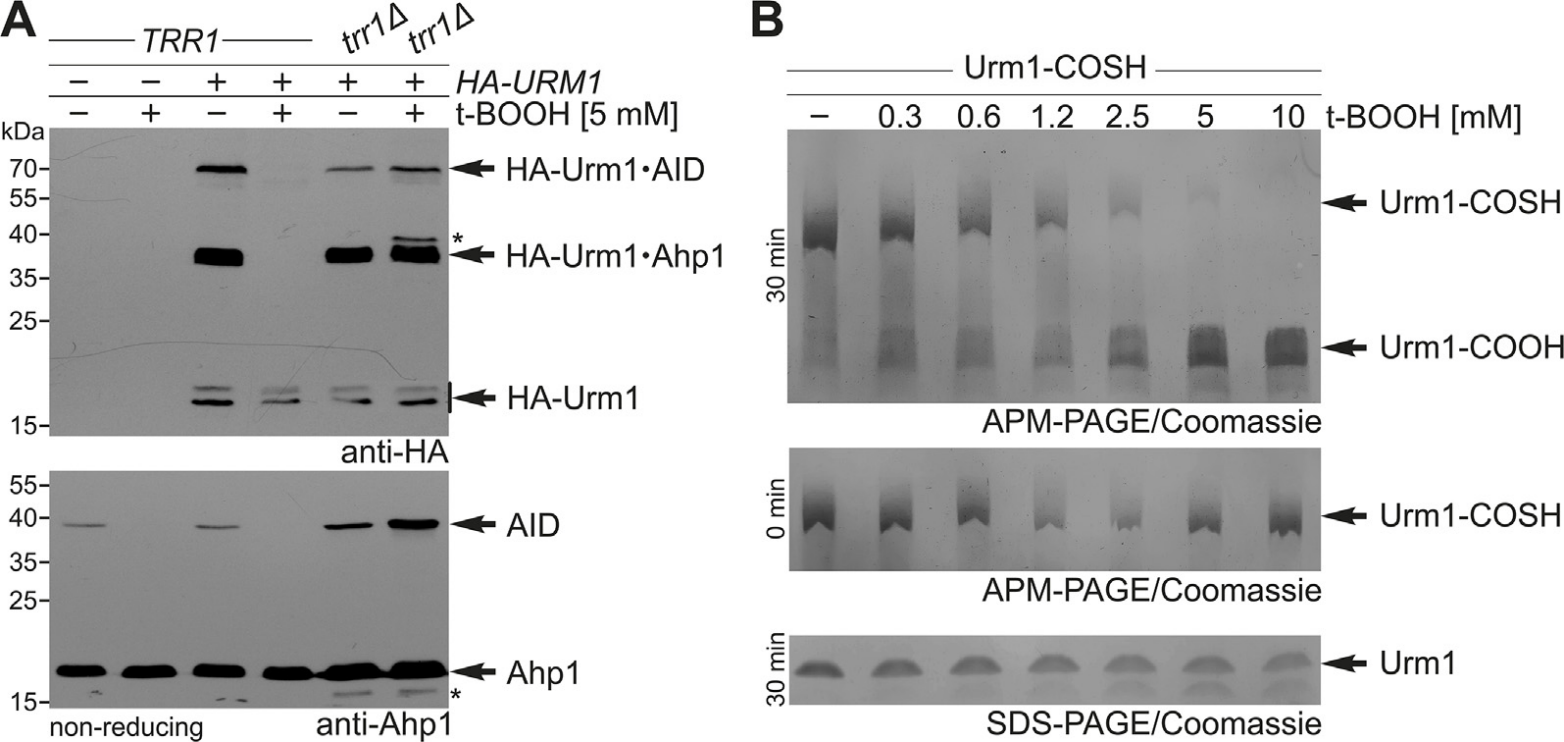
Effects of t-BOOH on Ahp1 reduction by thioredoxin and integrity of the thiocarboxylate of Urm1. (**A**) Inhibition of Ahp1 urmylation by t-BOOH relies on a functional thioredoxin reductase. EMSA under non-reducing conditions from *TRR1* and *trr1*Δ cells expressing *HA-URM1* (+) or not (−) in the presence (+) or absence (−) of 5 mM t-BOOH. HA-Urm1 conjugation was studied by anti-HA Western blot (top panel) diagnostic for free HA-Urm1 and urmylated forms of Ahp1 (~36 kDa) and Ahp1 intersubunit disulfides (AID ~72 kDa). The anti-Ahp1 blot (bottom panel) detects unmodified Ahp1 (~19 kDa) and AID (~38 kDa). Asterisks denote faster Ahp1 forms (bottom panel) in *trr1*Δ cells and an unknown anti-HA signal (top panel). (**B**) t-BOOH exposure of Urm1 *in vitro*. Recombinant Urm1-COSH was treated with indicated t-BOOH doses and analyzed under non-reducing conditions by APM gel electrophoresis at time-point 0 min (middle panel) and 30 min (top panel) and under reducing SDS-PAGE conditions in the absence of APM after 30 min (bottom panel). Arrows distinguish the thiocarboxylate (Urm1-COSH) from the inactive form (Urm1-COOH).

Prompted by data that exposure with the organic peroxide t-BOOH stimulates the formation of Ahp1 intersubunit disulfides [39], we next studied the impact of oxidative stress on Ahp1 urmylation *in vivo*. Using anti-HA and anti-Ahp1 EMSAs under reducing and non-reducing conditions, mild t-BOOH levels (0.3–0.6 mM) did not affect Ahp1 urmylation (Fig. 3). Intermediate t-BOOH doses (1.2–2.5 mM), however, progressively suppressed Ahp1 urmylation and formation of intersubunit disulfides, and highest doses (5–10 mM) eventually abolished both (Fig. 3). Thus, t-BOOH doses known to affect yeast cell growth *in vivo* [14] efficiently suppress Urm1 conjugation and Ahp1 disulfide formation (Fig. 3). Whether this involves t-BOOH interference with Ahp1, the thioredoxin system or Urm1-COSH, the thiocarboxylate critical for Ahp1 urmylation, is not known. In an effort to address these options, we found that lack of thioredoxin reductase Trr1 in the *trr1*Δ mutant counteracts the negative t-BOOH effect on urmylation seen with *TRR1* wild-type cells (Fig. 4A). As a result, HA-Urm1•Ahp1 conjugates and Ahp1 disulfides reappeared and even increased in relation to the untreated *trr1*Δ control (Fig. 4A). Thus, inhibition of urmylation by t-BOOH apparently relies on thioredoxin function.

Our data suggest Ahp1 hyperoxidation by t-BOOH *in vivo* since the intersubunit disulfides that are locked in the *trr1*Δ mutant (Fig. 4A) ought to resist oxidation. In line with this notion, excess t-BOOH previously led to conversion of the C_P_ thiol (Cys-62) into a sulfonate *in vitro* [12,41]. Hence, hyperoxidation may be ascribable, at least in part, for the inhibitory t-BOOH effects that we observe *in vivo* on urmylation of Ahp1 (Fig. 3). Alternatively, t-BOOH may interfere with Urm1-COSH, which is crucial for Ahp1 urmylation *in vivo* [19,21]. To study the latter, we exposed recombinant Urm1-COSH to t-BOOH and analyzed it by an APM-based gel retardation assay that distinguishes the starting material (Urm1-COSH) from the inactive form (Urm1-COOH) [42]. t-BOOH doses (1.2–2.5 mM) found to be effective *in vivo* (Fig. 3) gradually converted Urm1-COSH into mobile Urm1-COOH (Fig. 4B). At t-BOOH doses (5–10 mM) that abolished urmylation *in vivo* (Fig. 3), Urm1-COOH exclusively accumulated *in vitro* (Fig. 4B). With the latter being unable to urmylate proteins (including Ahp1) [19,21,29], our *in vitro* data (Fig. 4B) suggest that the t-BOOH effect *in vivo* (Fig. 3) may involve inactivation of Urm1. Collectively, high organic peroxide doses appear to prevent urmylation in yeast cells through a combination of negative effects on Urm1 and Ahp1.

### 2.4 The dimer interface is required for peroxidase and Urm1 acceptor activity of Ahp1

**Fig. 5:**
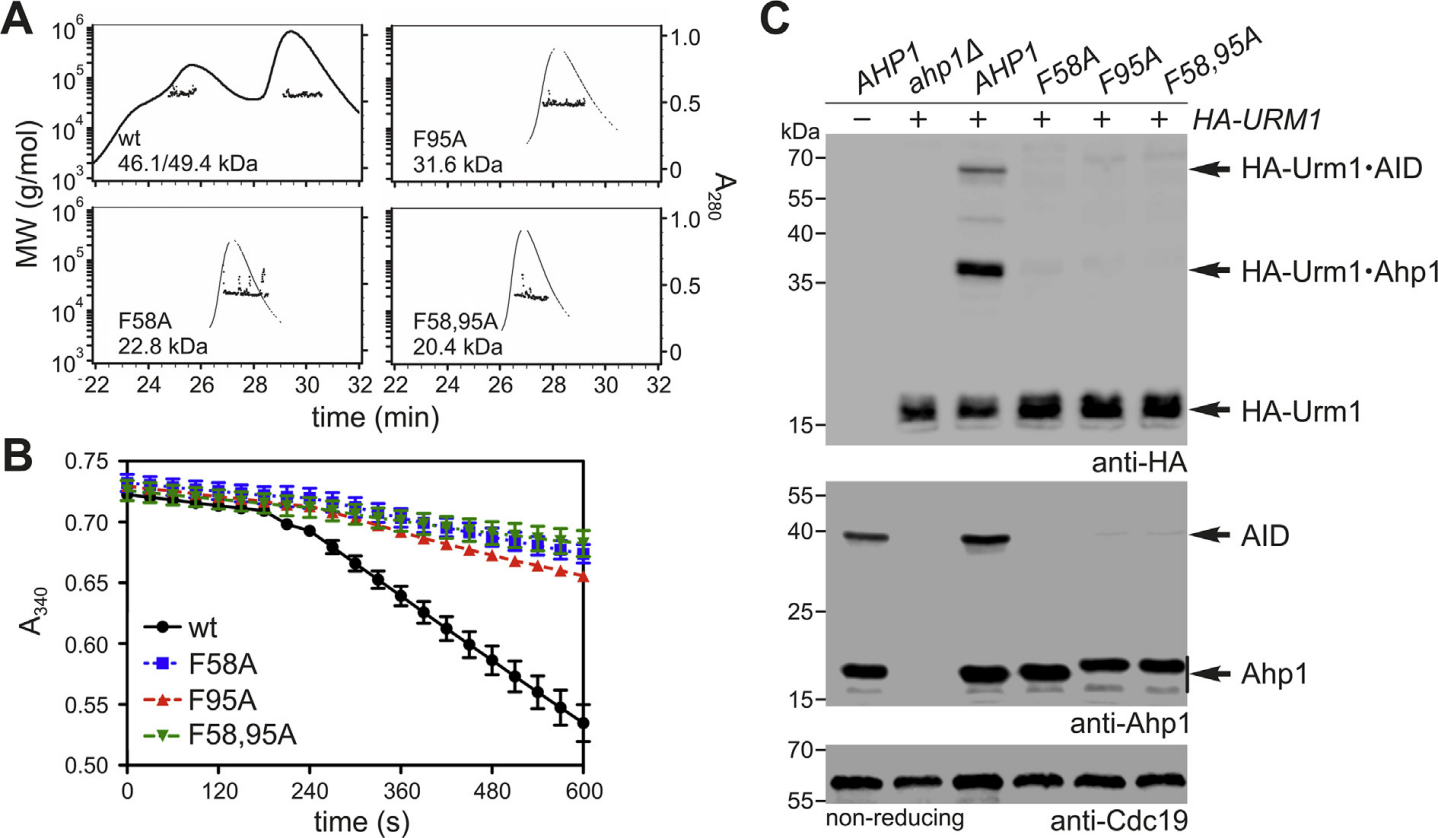
Ahp1 dimer interface mutations block peroxidase activity and urmylation. (**A**) Analysis of oligomeric state for Ahp1 variants by SEC-MALS. Traces represent A_280_ values of prominent peaks eluting off of the gel filtration column, and the scatter plotters underneath indicate the molecular weight (MW) ranges observed. The average molecular weight is given for the wild-type (wt) Ahp1 dimer and each interface mutant. (**B**) Coupled Ahp1 activity assays (see Materials & Methods). At 180 s, t-BOOH (100 μM) was added and NADPH absorbance at 340 nm monitored. An average of six independent measurements ± standard error of the mean is represented. (**C**) EMSA under non-reducing conditions from indicated strains expressing *HA-URM1* (+) or not (−). NEM-stabilized urmylation was studied by anti-HA blot (top panel) diagnostic for free HA-Urm1 and urmylated forms of Ahp1 (~36 kDa) and Ahp1 intersubunit disulfides (AID ~72 kDa). anti-Ahp1 blot (middle panel) detects unmodified Ahp1 (~19 kDa) and AID (~38 kDa). Protein loading control: anti-Cdc19 (bottom panel).

To investigate redox requirements of Ahp1 for Urm1 conjugation we asked whether urmylation is linked to dimerization of Ahp1. To do so, we resorted to structural data [14,15] showing that two conserved phenylalanine residues (Phe-58, Phe-95) (Fig. 1A and B) are located at the center of the dimer interface. When mutated (F58A; F95A; F58,95A), these were shown by native PAGE analysis to cause decreased dimerization [14]. To validate and extend the data, we estimated the molecular weights of wild-type Ahp1 and interface mutants via SEC-MALS (Fig. 5A), a technique coupling size exclusion chromatography with multiangle light scattering [43]. Despite a broad retention profile of wild-type Ahp1, the molecular weights for both distinguishable peaks via MALS analysis were in line with the value of an Ahp1 homodimer (46.7 kDa) (Fig. 5A).

**Fig. 6:**
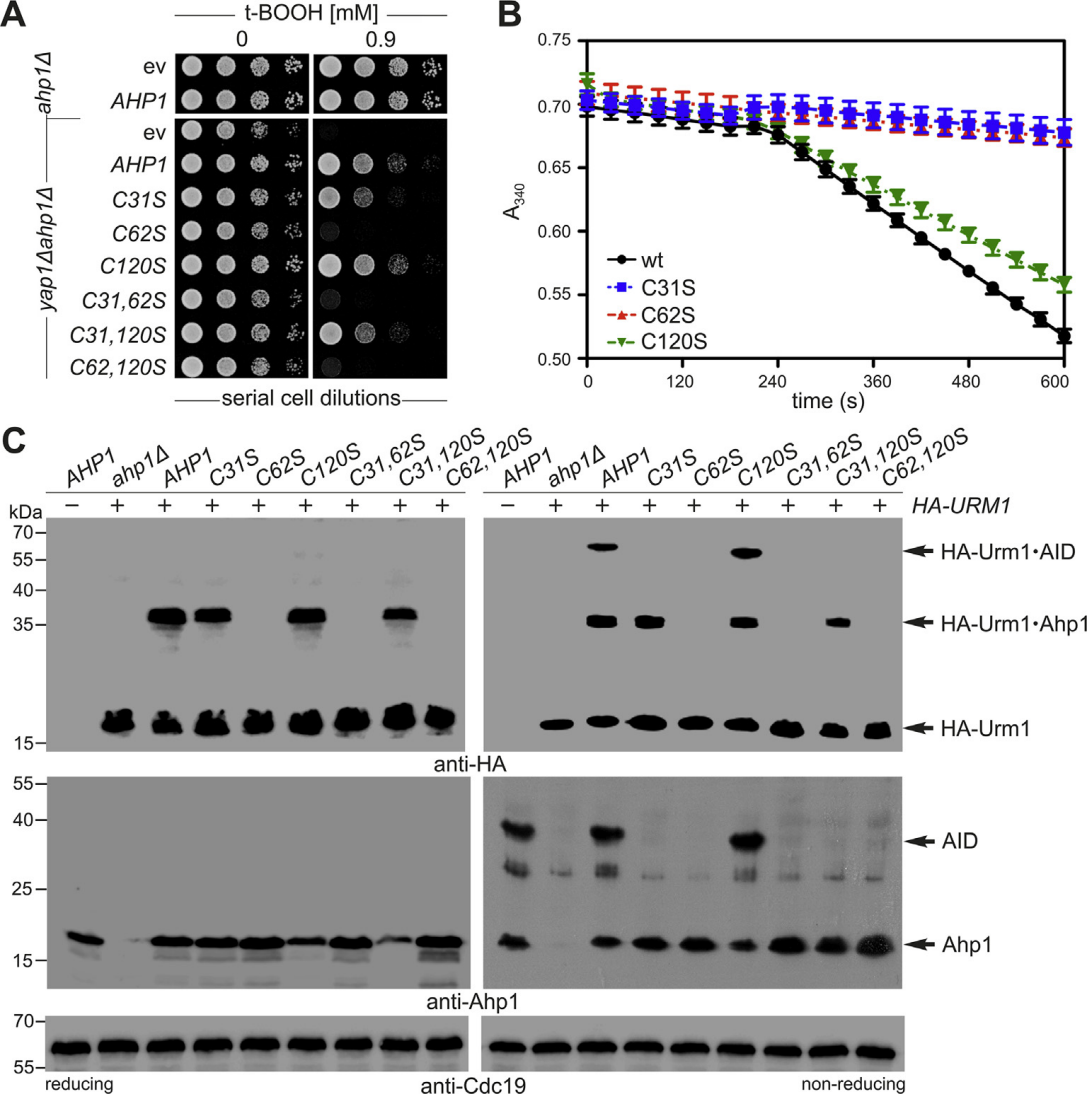
*AHP1* mutational analysis links Urm1 acceptor activity with peroxiredoxin function. (**A**) t-BOOH cytotoxicity assay *in vivo*. Growth of *ahp1*Δ or *yap1*Δ*ahp1*Δ cells carrying empty vector (ev), wild-type peroxiredoxin gene (*AHP1*) or indicated Cys substitutions was monitored without or with 0.9 mM t-BOOH. (**B**) Coupled Ahp1 activity assays. At 180 s, t-BOOH (100 μM) was added and NADPH absorbance at 340 nm monitored. An average of six independent measurements ± standard error of the mean is represented. (**C**) EMSA under reducing (left panels) and non-reducing (right panels) conditions from indicated genetic strain backgrounds expressing *HA-URM1* (+) or not (−). NEM-stabilized HA-Urm1 conjugation was studied by anti-HA blots (top panels) diagnostic for free HA-Urm1 and urmylated forms of Ahp1 (~36 kDa) or Ahp1 intersubunit disulfides (AID ~72 kDa). anti-Ahp1 Western blots (middle panels) solely detect unmodified Ahp1 (~19 kDa) and AID (~38 kDa). Western blots using anti-Cdc19 (bottom panels) served as internal protein standard.

In contrast, each of the interface mutants eluted at retention times comparable to wild-type Ahp1, yet as single peaks (Fig. 5A). However, all Ahp1 variants exhibited estimated molecular weights lower than that of a dimer, with most (except for F95A) approximately the value of a His-tagged Ahp1 monomer (~23.3 kDa) (Fig. 5A). In sum, our data confirm that substitution of subunit interface residues decreases the ability of Ahp1 to form dimers. Since the Ahp1 interface mutants disrupted oligomerization, we tested their peroxidase performance in a coupled activity assay with thioredoxin Trx2, thioredoxin reductase Trr1 and NADPH (Fig. 5B). Upon addition of t-BOOH, a sharp decrease in NADPH occurred for wild-type Ahp1 diagnostic for proper peroxide detoxification (Fig. 5B). In contrast, Ahp1 variants harboring single and double substitutions at the dimer interface resembled inactive enzymes lacking the crucial C_P_ or C_R_ thiols (C62S or C31S) (Fig. 6B).

Thus, our data show that the hydrophobic interface contributes to the ability of Ahp1 to form dimers and detoxify t-BOOH *in vitro*. In contrast to wild-type Ahp1, we observed by anti-HA and anti-Ahp1 EMSAs that the single (F58A; F95A) and double (F58,95A) interface mutants failed to be urmylated and lacked formation of Ahp1 intersubunit disulfides under non-reducing conditions (Fig. 5C). This shows an intact dimer interface is critical for anti-oxidant activity of Ahp1 and Urm1 conjugation. In line with this, the Phe-95 substitution alone or in tandem with the Phe-58 mutation were reported to enhance the sensitivity of yeast cells to growth inhibition by t-BOOH *in vivo* [14]. Together, our data demonstrate that dimerization and peroxidase activity are intimately linked with urmylation of Ahp1.

### 2.5 Mutagenesis of the redox-active thiol center in Ahp1 abolishes urmylation

Ahp1 was shown to be urmylated at Lys-32 close to its redox-active center (Cys-31 Cys-62) (Fig. 1B) [12,15,16,21]. Another cysteine residue (Cys-120) was assumed to be catalytic [16] before being refuted [15]. We examined whether serine substitution mutations at Cys-31, Cys-62 or Cys-120 would affect the sensitivity of an *ahp1Δ* null-mutant towards t-BOOH *in vivo*. Expression of C31S and C62S mutants failed to restore t-BOOH protection in *ahp1Δ* cells, while Ahp1 wild-type and the C120S mutant allowed for growth at t-BOOH doses of up to 2 mM (Suppl. Fig. S2). Yeast lacking Ahp1 and the oxidant-sensitive transcription factor Yap1 (*yap1Δahp1Δ*) are more sensitive to t-BOOH [39] than *ahp1Δ* cells. Expression of the C62S mutant in this background failed to protect against 0.9 mM t-BOOH, a dose tolerated by the C31S mutant (Fig. 6A). Hence, in the absence of Yap1, the importance of the C_R_ and C_P_ thiols for peroxidase activity apparently differs. Since t-BOOH tolerance was eliminated after substituting both redox-active thiols (C31,62S) in the double mutant (Fig. 6A), partial peroxidase activity seen with the C31S mutant alone depends on an active C_P_ (Cys-62). Therefore, unlike Cys-31, Cys-62 is critical for anti-oxidant function of Ahp1. The C120S mutant, however, did not noticeably alter t-BOOH sensitivity (Fig. 6A) and combined with the C_R_ or C_P_ mutations (C31,120S or C62,120S), there are no additional growth defects compared to the single C31S or C62S mutants alone (Fig. 6A) (Suppl. Fig. S2).

Our *in vivo* data are in agreement with *in vitro* Ahp1 peroxidase activity assays (Fig. 6B). Upon addition of t-BOOH, we observed a marked decrease in NADPH indicative for peroxide detoxification by wild-type Ahp1 and the C120S mutant, while peroxidase activity with C31S or C62S was negligible (Fig. 6B). In addition, we found wild-type like urmylation levels including formation of urmylated (or non-modified) Ahp1 intersubunit disulfides in the Cys-120 mutant (Fig. 6C). Next, we asked whether C_R_ (C31S) and/or C_P_ (C62S) substitutions would affect Ahp1 oxidation and urmylation. As judged from anti-Ahp1 EMSA, the Cys-31 and Cys-62 substitutions alone (C31S; C62S) or in combination (C31,62S; C31,120S; C62,120S) all failed to form Ahp1 intersubunit disulfides under non-reducing conditions (Fig. 6C). This agrees with our data (Fig. 6A and B) showing that the C_R_ and C_P_ thiols are key to the Ahp1 peroxidatic cycle (Fig. 1B). However, based on anti-HA EMSA, each mutation behaved different in terms of urmylation (Fig. 6C). While single C31S and double C31,120S mutants formed Urm1 conjugates under reducing and non-reducing conditions, C62S failed to do so.

This indicates that disulfide formation upon oxidation is dispensable for urmylation, whereas the C_P_ thiol (Cys-62) is essential for the conjugation reaction (Fig. 6C). Accordingly, when combined with C31S or C120S, the negative C62S effect dominates causing loss of urmylation in each double mutant (C31,62S or C62,120S) (Fig. 6C). Thus, Cys-62 is essential for urmylation even in the case of the C31S mutant, which lacks disulfide formation upon oxidation by the peroxide and cannot be recycled. This finding indicates that oxidation of Cys-62 rather than disulfide formation upon Cys-62 oxidation is critical for urmylation.

### 2.6 Analysis of lysine-based acceptor sites for urmylation of Ahp1

**Fig. 7:**
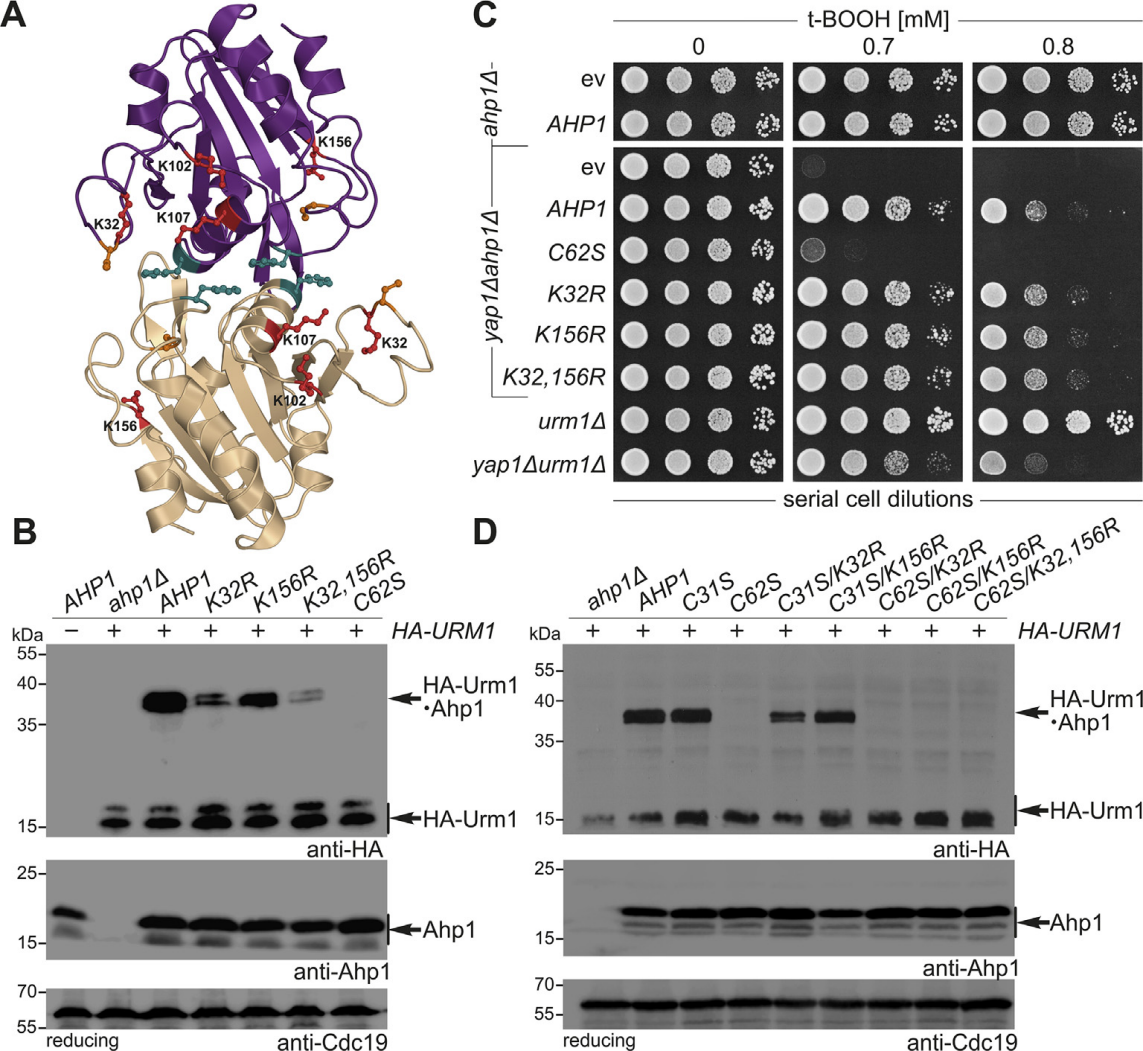
Lysine-directed Ahp1 urmylation *in vivo* requires the catalytic C_P_ thiol (Cys-62). (**A**) Overview of lysine residues (K32, K102, K107, K156) close to the redox-active center in the reduced form of the Ahp1 homodimer (see Fig. 1A and B). (**B, D**) Shown are EMSAs under reducing conditions from the indicated strains expressing *HA-URM1* (+) or not (−). NEM-stabilized HA-Urm1 conjugation was studied by anti-HA blot (top panels) diagnostic for free HA-Urm1 and urmylated Ahp1 (~36 kDa). anti-Ahp1 Western blot (middle panels) detects unmodified Ahp1 (~19 kDa); anti-Cdc19 (bottom panels) served as internal standard. (**C**) t-BOOH cytotoxicity assay *in vivo*. Growth of *ahp1Δ* or *yap1Δahp1Δ* cells carrying empty vector (ev), wild-type peroxiredoxin gene (*AHP1*) and cysteine or lysine substitutions was monitored together with *urm1Δ* and *yap1Δurm1Δ* reference cells without or with the indicated t-BOOH doses.

To monitor a possible link between the redox-active center in Ahp1 and lysine-directed Urm1 conjugation, we studied the impact of lysine substitutions in Ahp1 on urmylation. The proximity of Lys-32 and Lys-156 (Fig. 7A) to the catalytic thiols in the crystal structure of Ahp1 [15] prompted us to generate mutants with both replaced by arginine alone or in combination (K32R; K156R; K32,156R). We observed slightly decreased urmylation levels in the K156R mutant compared to wild-type suggesting a minor target role (Fig. 7B).

Unlike previously reported [21], urmylation in the K32R mutant was not entirely abolished, but was significantly decreased compared to wild-type or K156R cells (Fig. 7B). This indicates Lys-32 is targeted more easily by Urm1 than Lys-156, yet it is not essential to provide Ahp1 with full Urm1 acceptor activity. Strikingly, the absence of both residues in the double mutant (K32,156R) enhanced the urmylation defects of each single mutant (K32R or K156R) (Fig. 7B). As a result, Urm1 conjugation dropped to significantly low levels, albeit not as dramatic as with complete loss of urmylation seen in the peroxidase-dead mutant (C62S) (Fig. 7B). This additive negative effect suggests that Lys-156 is an alternative urmylation site, particularly when Lys-32 is unavailable due to a substitution mutation. Moreover, based on low residual urmylation left in the double mutant (K32,156R) the existence of other Urm1 target sites has to be assumed.

Hence, we consider lysine-directed Urm1 conjugation to Ahp1 may be promiscuous and less specific to Lys-32 than originally [21] anticipated. Urmylation at lysine residues next to the catalytic thiols may interfere with the anti-oxidant activity of Ahp1. Therefore, we examined whether the K32R, K156R and K32,156R mutants would affect t-BOOH sensitivity *in vivo* in relation to peroxidase-minus (C62S) or *URM1* and *YAP1 URM1* deletion strains (Fig. 7C). In the *yap1Δahp1Δ* strain, the K32R, K156R and K32,156R mutants all restored t-BOOH tolerance similar to that of wild-type *AHP1* (Fig. 7C). This is in contrast to the peroxidase-dead mutant (C62S), which fails to change the t-BOOH sensitivity and lacks urmylation (Fig. 7B and C). Together with t-BOOH sensitivity of the *yap1Δurm1Δ* double mutant (Fig. 7C), our data thus indicate that the lysine substitution mutants do not significantly differ in their response to t-BOOH cytotoxicity from *yap1Δ* cells that express wild-type Ahp1 but cannot undergo protein urmylation due to *URM1* gene deletion. Hence, lysine dependent urmylation defects (K32R; K32,156R) appear not to affect the anti-oxidant activity of Ahp1.

In agreement with our mutational analysis of the redox-active cysteines above (Fig. 6), we confirmed that for lysine-directed Urm1 conjugation to occur, Ahp1 must be catalytically active (Fig. 7D). Thus, residual urmylation levels typical of the lysine substitution mutants (K32R; K156R; K32,156R) (Fig. 7B) were found to be abolished upon mutation of the C_P_ thiol (C62S) rather than the C_R_ thiol (C31S) (Fig. 7D). In further support of this view are studies with the human homolog of Urm1 (hURM1), which we had previously shown to modify Ahp1 in yeast [20]. Here, our data show that, as is the case with yeast Urm1, the ability of hURM1 to form lysine-directed conjugates depends on the integrity of the C_P_ thiol (Cys-62) and hence, on the anti-oxidant activity of Ahp1 (Suppl. Fig. S3).

## 3 Discussion

Unlike conventional ubiquitin-like proteins (e.g. SUMO, Nedd8, UFM1), Urm1 undergoes activation by C-terminal thiocarboxylation (Urm1-COSH) [29,44,45]. Thus thiolated Urm1 is dual-functional, engaging in two sulfur-dependent modification pathways, namely tRNA thiolation and ubiquitin-like Urm1 conjugation [[46], [47], [48], [49]]. While the former function resembles bacterial sulfur carriers (e.g. ThiS-COSH or MoaD-COSH) engaged in thio-cofactor synthesis (e.g. thiamin or molybdopterin) [[46], [47], [48],^50^,^51^], the latter involves oxidant-induced, lysine-directed protein urmylation in eukaryotes [17,21,52]. Similarly, prokaryotic Urm1-like proteins (e.g. TtuB, SAMP1-SAMP3) with dual modification functions have been identified, and Urm1-like conjugation including archaeal sampylation can be triggered by oxidants, too [[19], [20], [21],^32^,^33^]. This suggests a conserved function of Urm1 family members in oxidative stress responses, and indeed among identified Urm1 targets from yeast, fungi, flies and human cells are 2-Cys peroxiredoxins (Ahp1, Prx5) [[18], [19], [20], [21], [22],^32^,^33^].

The redox requirements of Ahp1 and molecular determinants that influence its urmylation are largely unknown. Our analyses of unmodified and urmylated Ahp1 under reducing or non-reducing conditions show that only a fraction of Ahp1 is subject to urmylation *in vivo*. Thus, under our experimental conditions, Ahp1 urmylation is not limiting, a notion in accordance with quantitative proteomic studies showing that in budding yeast, Ahp1 is considerably more abundant than Urm1 [53]. In contrast to canonical ubiquitination [54], we find no evidence for oligo- or poly-urmylation, and among the urmylated pool are Ahp1 intersubunit disulfides, which carry one Urm1 copy attached to each subunit. Since we are not aware of a deurmylase activity from yeast (or other model systems), it remains to be elucidated whether Urm1 is permanently attached to Ahp1.

In addition, we show that treatment of yeast cells with high t-BOOH concentrations decreases urmylation of Ahp1 (Fig. 3). This negative effect of t-BOOH on urmylation requires a proper thioredoxin system (Trr1, Trx1, Trx2; Fig. 2, Fig. 4). In the absence of cytosolic thioredoxins or thioredoxin reductase, Ahp1 should be more frequently in the disulfide-linked state, thereby preventing hyperoxidation. Consistent with this notion, peroxiredoxins that more quickly resolve to form the disulfide upon sulfenic acid formation are more resistant to hyperoxidation [55,56]. In contrast, continuous reduction by thioredoxin may increase the chance of hyperoxidation, as is the case for other peroxiredoxins [39,[55], [56], [57], [58], [59], [60]]. The anti-oxidant activity of Ahp1 requires a constitutive homodimer, in which two subunits generate the 2-Cys-based redox-active (Cys-31 Cys-62) centers (Fig. 1A and B) [14,15,43]. Assembly of active peroxiredoxin through a well-defined hydrophobic subunit interface in the Ahp1 dimer (Phe-58, Phe-95) (Fig. 1A and B) [14] apparently is a prerequisite for Urm1 conjugation *in vivo*. Hence, interface mutants of Ahp1 (F58A; F95A; F58,95A) which no longer form dimers fail to be urmylated, even in the presence of the catalytic Cys residues (Fig. 5C). Nevertheless, substituting the catalytically critical C_P_ thiol (C62S) alone or in tandem with the C_R_ thiol (C31,62S) or a third thiol (C62,120S) leads to peroxidase-dead enzymes that lack urmylation (Fig. 6C). Hence, the integrity of Cys-62, which upon sulfenylation by t-BOOH forms with Cys-31 the intersubunit disulfides (Fig. 1B) [15,16,57,59], is essential for both peroxide detoxification and Ahp1 urmylation.

Despite such strict dependence on the C_P_ thiol (Cys-62), Ahp1 urmylation after all results in ubiquitin-like, lysine-directed protein conjugation [21,61]. Given that Cys residues can be ubiquitinated [[62], [63], [64]], primary Urm1 transfer onto Ahp1 may involve a non-lysine site (i.e. Cys-62) followed by subsequent lysine-directed iso-peptide linkage. If so, it will be important to delineate the redox state of Cys-62 required for Urm1 transfer. That oxidation may be important is supported by data showing thiol-active agents (H_2_O_2_, t-BOOH, diamide, NEM) trigger urmylation of proteins including peroxiredoxins Prx5 and Ahp1 [19–21,32,33].

**Fig. 8:**
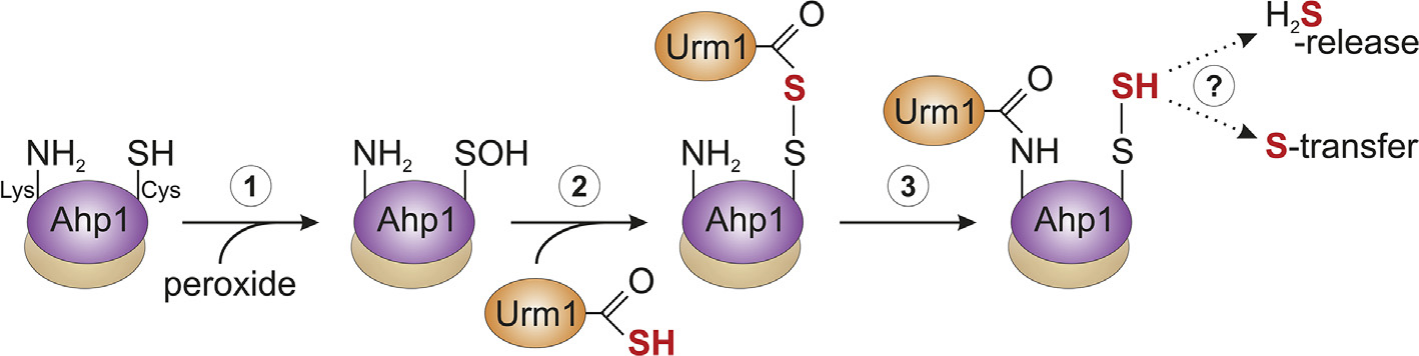
Working model for ubiquitin-like urmylation of yeast peroxiredoxin Ahp1. Step 1: The peroxidatic thiol of Ahp1 (Cys-SH) reacts with a peroxide to form a sulfenic acid (Cys-SOH), which following the fully folded to locally unfolded (FF-LU) transition [59] may become surface exposed. Step 2: The sulfenic acid (Cys-SOH) condenses with the activated thiocarboxylate of Urm1 (Urm1-COSH) to form an acyl disulfide (Ahp1-S-S–CO–Urm1). Step 3: The ε-amino group of a nearby Lys residue mounts a nucleophilic attack on the carbonyl group of the acyl disulfide generating an iso-peptide bond (Lys–NH–CO-Urm1) between Ahp1 and Urm1 and a persulfidated cysteine (Cys-S-SH). Whether Lys-based urmylation stabilizes the persulfide on Ahp1, triggers H_2_S-release or drives *trans*-persulfidation of other targets is not known. For simplicity, the mechanistic hypothesis involves only one subunit of the Ahp1 homo-dimer.

In principle, the thiolate that Cys-62 forms in its reduced state may react with the thiocarboxylate of Urm1 (Urm1-COSH) to produce a thioester. However, taking into account that in its reduced state, Ahp1 is fully folded [12,65], the thiol of Cys-62 may not be accessible without local unfolding for Urm1-COSH transfer. C_P_ thiol (Cys-62) oxidation and sulfenylation (-SOH) by peroxide (Fig. 8) could facilitate unfolding and prime the formation of an acyl disulfide between Ahp1 and Urm1 (Ahp1-S-S–CO–Urm1) rather than the above thioester. From organic chemistry *in vitro* it is known that acyl disulfides, which form between the thiocarboxylate of one peptide and an activated thiol of a second carrying a free amino group, are short-lived and readily ligate via iso-peptide bonds [[66], [67], [68]]. In analogy, we envision that the acyl disulfide (Ahp1-S-S–CO–Urm1) formed *in vivo* is highly reactive and undergoes a nucleophilic attack on its carbonyl group by the ε-amino group of a nearby lysine residue (Fig. 8). This will generate an iso-peptide bond with Urm1 (Ahp1-NH–CO–Urm1) and leave the peroxidatic cysteine persulfidated (Cys-S-SH) (Fig. 8). Whether Lys-based urmylation stabilizes this persulfide on Ahp1 for *trans*-persulfidation of other targets is not known (Fig. 8).

In line with our working model (Fig. 8), a previous report [21] showed that among ten out of fourteen lysines tested for Ahp1 target site function, Lys-32 (next to Cys-31) is necessary for iso-peptide linkage with Urm1. However, we find that Lys-32 substitution alone (K32R) or in tandem with Lys-156 (K32,156R), which maps proximal to the C_P_ thiol (Cys-62) (Fig. 7A), still allows residual urmylation. So, rather than being essential, Lys-32 likely represents one of several target sites for Urm1 conjugation with Ahp1. In support of promiscuous lysine sites, which from conventional ubiquitylation substrates in yeast (e.g. Sic1, Rpn4) [[69], [70], [71]] are not unheard of, we observe a minor Urm1 target role for Lys-156, and two more lysine residues, Lys-102 and Lys-107, can be found proximal to the active site in Ahp1 (Fig. 7A).

Although Urm1 is conserved in eukaryotes [20,34], the precise role the modifier plays for its target proteins is ill-defined. As for Ahp1, we have shown here that substitutions of the lysine-based urmylation sites (K32R; K32,156R) exhibit negligible effects on the anti-oxidant activity of the enzyme *in vivo* while peroxidase-dead mutants (C62S; C31,62S; C62,120S) all fail to be urmylated. Nonetheless, Urm1 attachment occurs close to the redox-active (Cys-31 Cys-62) center (Fig. 1A and B) and near the Ahp1-Trx2 interface [15], which is why urmylation cannot be excluded to interfere with some aspect of the Ahp1 peroxidatic cycle (e.g. t-BOOH detoxification or thioredoxin reduction; Fig. 1B). In support, Ahp1 regeneration by Trx2 has been shown to be affected in Lys-32 substitution mutants (K32A; K32E) *in vitro* [15]. Bearing in mind that a bacterial sulfur carrier with an Urm1-like fold (CysO-COSH) is more resistant to oxidation than sulfide and upregulated by oxidative stress [72], the role for yeast Urm1-COSH may also relate to peroxides, in particular t-BOOH, the preferred substrate of Ahp1 [11,12]. The latter notion agrees with our *in vivo* studies showing that while Urm1 conjugation was detectable in response to mild and intermediate t-BOOH doses, higher peroxide levels gradually prevented urmylation and significantly decreased Ahp1 disulfide formation.

We cannot exclude that inactivation by t-BOOH of Ahp1 or Urm1-COSH itself may impede urmylation of the peroxiredoxin *in vivo*. However, Ahp1 has been co-purified with a sulfiredoxin (Srx1) capable to reduce hyperoxidized Tsa1 [73], a distantly related 2-Cys peroxiredoxin [43]. Moreover, *in vitro* we found indications for peroxide inactivation of Urm1-COSH (Fig. 4B) suggesting this may constitute a factor accounting for the negative t-BOOH effects on Ahp1 redox biology and urmylation (Fig. 3). In sum, our comprehensive urmylation analysis of the 2-Cys peroxiredoxin Ahp1 has laid the foundation to better understand the redox requirements for Urm1 conjugation *in vivo* and provide insight into the mechanism for urmylation of other protein targets.

## 4 Material and methods

### 4.1 Yeast strains, plasmid constructions and general methods

Growth of yeast strains (Table S1) was in routine YPD or SC media [74] for 3 days at 30 °C. Primers used for PCR-based protocols [[75], [76], [77], [78]] to generate and diagnose site-specific *AHP1* mutations, gene deletions or epitope tagged gene fusions are listed in Table S2. PCR-based site-directed mutagenesis (SM-PCR [79]) for generation of Ahp1 cysteine to serine (C31S, C62S, C120S), phenylalanine to alanine (F58A, F95A) and lysine to arginine (K32R, K156R) substitutions or double combinations thereof (C31,62S; C31,120S; C62,120S; F58,95A, K32,156R, C31S/K32R, C31S/K156R, C62S/K32R, C62S/K156R, C31S/K32,156R) was as previously described using single-copy expression plasmids [80] (Table S3) and verified by Sanger based DNA sequencing. For *AHP1* expression in yeast, the ORF of *AHP1* and variants were amplified by PCR from chromosomal DNA of yeast strain BY4741 (Table S1) and cloned into YCplac111 generating pAJ31. Epitope-tagged *AHP1-(c-myc)*_*9*_ was amplified from chromosomal DNA of strain FEY14 [20] (Table S1) and cloned into YCplac111 to generate pAJ19. Transformation of yeast cells with PCR products or plasmids [80,81] was done as previously described [82,83]. t-BOOH toxicity assays *in vivo* involved BY4741, *ahp1Δ* or *ahp1Δyap1Δ* strains transformed with empty vector (YCplac111) or Ahp1 expression constructs (Table S1). These were diluted to an OD_600_ of 1.0 from which 10-fold serial dilutions were spotted onto YPD plates in the absence or presence of 0.6–2.0 mM t-BOOH (*tert*-butyl hydroperoxide, Sigma) and grown for 36–48 h at 30 °C.

### 4.2 Urmylation studies using electrophoretic mobility shift assays (EMSA)

Urmylation studies were done essentially as described [20] with yeast grown in standard SC media at 30 °C to an OD_600_ of ~1.0. Cell lysis was done with a bead beater in a buffer (10 mM K-HEPES pH 7.0, 10 mM KCl, 1.5 mM MgCl_2_, 0.5 mM PMSF, 2 mM benzamidine, complete protease inhibitors (Roche) and 10 mM N-ethylmaleimide (NEM)) as previously described [18,20]. In some experiments, the cell culture was pre-incubated with t-BOOH (0.3–10 mM) for 5 min immediately before lysis. Following centrifugation at 16.000*g*, protein concentration in the supernatant was determined according to Bradford [84]. The lysates were mixed with sample buffer (62.5 mM Tris-HCl pH 6.8, 2% SDS, 10% glycerol, 0.002% bromophenol blue and ±5% β-mercaptoethanol) according to Lämmli [85], subjected to SDS-PAGE and transferred onto PVDF membranes. For EMSAs and Western blot analyses, PVDF membranes were incubated with primary anti-HA antibodies (F7, Santa Cruz Biotechnology or 2–2.214 Invitrogen). Unconjugated Ahp1 samples were probed with anti-Ahp1 serum [39] kindly provided by Dr Kuge (Tohoku Pharmaceutical University, Japan) and horseradish peroxidase-conjugated secondary goat anti-mouse or anti-rabbit IgGs (Jackson ImmunoResearch). Protein loading was checked using anti-Cdc19 antibodies provided by Dr Thorner (University of California-Berkeley, USA).

### 4.3 Expression and production of thiocarboxylated Urm1

In order to obtain thiocarboxylated Urm1, the Urm1-Intein-CBD-His_6_ fusion protein was overexpressed in *E. coli* and purified according to Refs. [29,86] with modifications. In brief, the bacterial pellet was resuspended in lysis buffer without reducing agent and lysed to homogeneity. The lysate was passed through a Ni-NTA column and, following washes, the fusion protein was eluted with elution buffer (30 mM Tris-HCl pH 7.5; 300 mM NaCl; 250 mM imidazole and 10% glycerol). The eluates were dialyzed overnight to chitin column buffer (30 mM Tris-HCl pH 8 and 500 mM NaCl) and applied on a chitin column. The column was washed with chitin column buffer and the cleavage of the tag was induced through incubation with cleavage buffer (30 mM Tris-HCl pH 8; 500 mM NaCl and 35 mM ammonium sulfide) for 16 h at room temperature. This procedure leads to the formation of Urm1 without additional residues at the N-terminus and with a thiocarboxylated C-terminal glycine (Urm1-COSH). The eluted Urm1-COSH was further purified by size-exclusion chromatography on a HiLoad 16/600 Superdex 75 column on ÄKTA™ start system and stored at −80 °C in storage buffer (20 mM Tris pH 7.5 and 200 mM NaCl). The presence of thiocarboxylated C-terminus was confirmed by running the protein on a polyacrylamide gel containing 25 μM APM ([N-Acryloyl-amino] phenyl) mercuric chloride [21,42].

### 4.4 Bacterial protein expression and purification of Ahp1

Procedures for cloning bacterial expression constructs for Trx2 and Ahp1 in the vector pET45b have been reported previously [14,87]. The Trr1 was amplified out of *S. cerevisiae* genomic DNA (Primer listed in Table S2). The PCR product was digested with *Nde*I and *Xho*I and subsequently cloned into pET29a with a C-terminal His tag. All clones were validated by DNA sequencing. Procedures for expressing and purifying His-tagged Ahp1 and Trx2 proteins have been reported previously [14,88]. A similar procedure was followed for the expression and purification of Trr1. Briefly, *E. coli* Rosetta cells transformed with pET29a-Trr1 were grown to mid-log phase in 400 mL LB medium containing 100 μg/mL ampicillin. Trr1 expression was induced with 1 mM IPTG for 6 h at 37 °C. Proteins were purified from cell pellets using the Qiagen NiNTA Fast-Start kit. Eluted proteins were desalted using PD Minitrap G25 column equilibrated with TDG buffer (50 mM Tris pH 7.5, 2 mM DTT, 10% glycerol and protease inhibitor cocktail (G Biosciences)). Proteins were estimated to be >95% pure by reducing SDS-PAGE. Extinction coefficients for proteins were estimated from the protein coding sequences as follows: Ahp1 (37950 M^−1^ cm^−1^), Trx2 (18,020 M^−1^ cm^−1^), and Trr1 (24719 M^−1^ cm^−1^).

### 4.5 Analysis of oligomeric state of Ahp1-dimer interface variants

Size exclusion chromatography coupled with multiangle light scattering (SEC-MALS) was used to monitor oligomeric state of Ahp1 proteins. Briefly, proteins were reduced in TDG by adding additional DTT to a final concentration of 50 mM and incubating for 30 min at room temperature. Reduced proteins were exchanged into SEC-MALS buffer (20 mM HEPES pH 7.5, 100 mM NaCl and 1 mM TCEP) using a BioSpin 6 column and diluted to 125 μM. Proteins were resolved on a gel filtration column and analyzed by MALS as previously described [43].

### 4.6 Analysis of Ahp1 peroxidase activity

Ahp1 variants (50 μL) were reduced with 50 mM DTT for 1 h at room temperature, prior to buffer exchange into 50 mM HEPES (pH 8.0) using a Biospin 6 column (BioRad). Activity assays were conducted using a coupled activity assay with t-BOOH, Trx2, Trr1 and NADPH [15]. Briefly, assays were conducted in 50 mM HEPES (pH 8.0) containing 0.5 μM Ahp1, 0.5 μM Trx2, 0.5 μM Trr1, and 125 μM NADPH. Prior to t-BOOH addition, a baseline reading was obtained every 30 s. At 180 s, 100 μM t-BOOH was added, and reactions were monitored at A_340_ every 30 s for 420 s.

### 4.7 In vitro t-BOOH response assay

500 ng of thiocarboxylated Urm1 was mixed in reaction buffer (20 mM Tris pH 7.5 and 200 mM NaCl). 0–10 mM tBOOH, was included and excluded as indicated. The reaction mix was incubated for 30 min at 30 °C, stopped by adding Lämmli sample buffer ± DTT and incubated for 5 min at 95 °C. Subsequently the thiocarboxylated Urm1 samples were loaded on SDS-PAGE gel containing 25 μM APM or not. For protein visualization, the gels were stained with Coomassie Brilliant Blue.

## Declaration of competing interest

The authors declare that they have no known competing financial interests or personal relationships that could have appeared to influence the work reported in this paper.
