# Supplementary material for: Redox requirements for ubiquitin-like urmylation of Ahp1, a 2-Cys peroxiredoxin from yeast
Source: Redox Biol. 2020 Jan 22;30:101438. doi: 10.1016/j.redox.2020.101438 (PMC7016264; doi:10.1016/j.redox.2020.101438)
Supplement: Multimedia component 1 [file mmc1.pdf]

Supplemental Material

Redox requirements for ubiquitin-like urmylation of Ahp1, a 2-Cys peroxiredoxin from yeast

Cindy Brachmann[a,§], Lars Kaduhr [a,§], André Jüdes[a,§,], Keerthiraju Ethiraju Ravichandran[b,c], James D. West[d], Sebastian Glatt[b] and Raffael Schaffrath[a,*]

[a] Universität Kassel, Institut für Biologie, Fachgebiet Mikrobiologie,
Heinrich-Plett-Str. 40, 34132 Kassel, Germany

[b] Max Planck Research Group at the Malopolska Centre of Biotechnology,
Jagiellonian University, 30-387 Krakow, Poland

[c] Postgraduate School of Molecular Medicine, 02-091Warsaw, Poland

[d] Biochemistry & Molecular Biology Program, Departments of Biology and Chemistry,
The College of Wooster, Wooster, OH, USA

[§] These authors contributed equally to this study

***** corresponding author:

Prof. Dr. Raffael Schaffrath
Phone: +49-561-804-4175
FAX: +49-561-804-4337
eMail: schaffrath@uni-kassel.de

1) Supplemental Figures

Fig. S1.

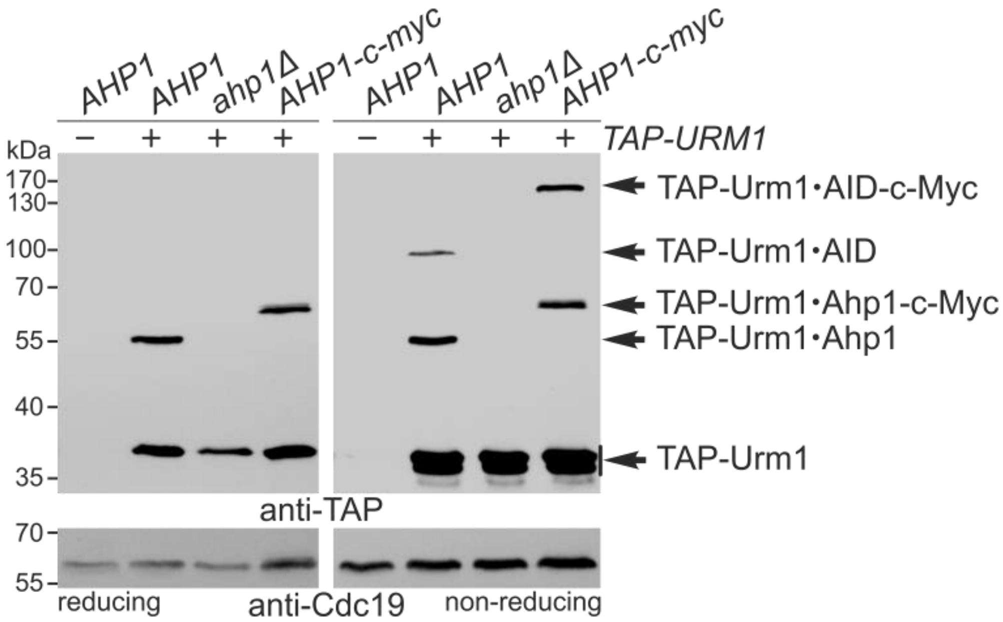

Supplemental Fig. S1. TAP-Urm1•Ahp1 conjugation *in vivo*. Shown are EMSAs under reducing (left panels) and non-reducing (right panels) conditions with protein extracts from indicated strains expressing *TAP-URM1* (+) or not (-). NEM-stabilized urmylation was studied with anti-TAP (top panels) diagnostic for free TAP-Urm1 (~37 kDa), urmylated Ahp1 (~55 kDa) and Ahp1 intersubunit disulfide (AID ~100 kDa) forms as well as Urm1-modified forms of c-Myc tagged Ahp1 (~65 kDa) and AIDs (~135 kDa). anti-Cdc19 blots (bottom panels) served as loading control. Reproduced in part from permission by Jüdes *et al.* (2016): *Microb Cell* 3: 554-564 (doi: 10.15698/mic2016.11.539).

Fig. S2.

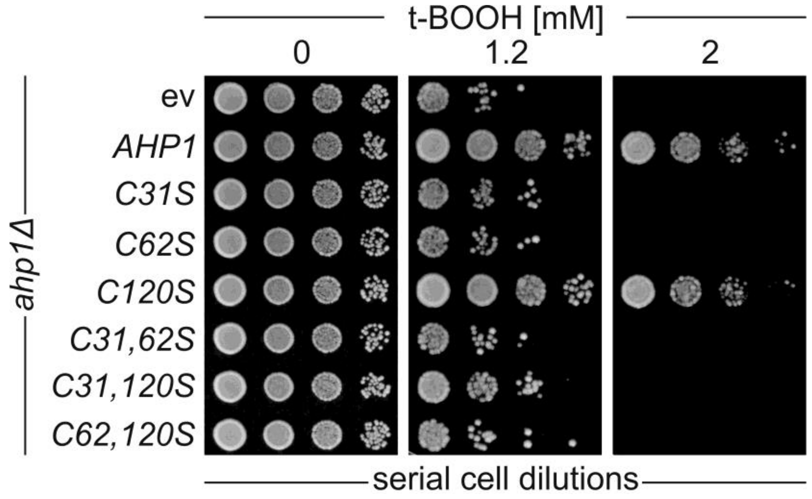

Supplemental Fig. S2. t-BOOH cytotoxicity assay *in vivo*. Using ten-fold serial cell dilutions, growth of an *ahp1*Δ yeast strain carrying empty vector (ev), wild-type peroxiredoxin (*AHP1*) gene or indicated Cys substitutions of Ahp1 (C31S; C62S; C120S; C31,62S; C31,120S; C62,120S) was monitored at 30°C in the presence of the denoted t-BOOH doses.

Fig. S3.

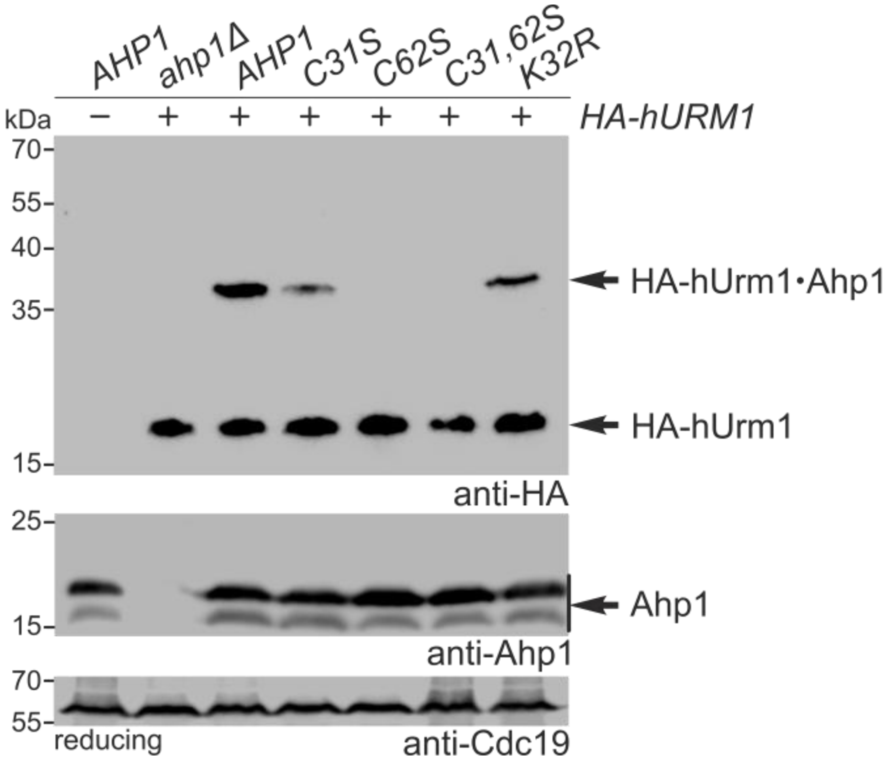

Supplemental Fig. S3. Ahp1 urmylation by the human homolog of yeast Urm1, hURM1, requires peroxidatic Cys-62. Shown are EMSA under reducing conditions from indicated strains expressing human HA-tagged hURM1 (+) or not (-). NEM-stabilized HA-hURM1 conjugation was studied by anti-HA blot (top panel) diagnostic for free HA-hURM1 and urmylated Ahp1 (~36 kDa). anti-Ahp1 (middle panel) detects unmodified Ahp1 (~19 kDa) and anti-Cdc19 (bottom panel) served as internal standard.

2) Supplemental Tables

Supplemental Table S1. Yeast strains used in this study.

Strain	Genotype	Source
BY4741	*MATa his3Δ1 leu2Δ0 met15Δ0 ura3Δ0*	Euroscarf
Y01400	BY4741, *urm1Δ::kanMX4*	Euroscarf
Y02720	BY4741, *ahp1Δ::kanMX4*	Euroscarf
FEY14	BY4741, *urm1Δ::kanMX4 AHP1-c-myc::ScHIS3*	[20]
FEY16	BY4741, *ahp1Δ::kanMX4 urm1Δ::ScHIS3*	[20]
FEY18	BY4741, *yap1Δ::kanMX4 ahp1Δ::SpHIS5*	This study
FEY42	BY4741, *urm1Δ::kanMX4 trx1Δ::SpHIS5*	This study
FEY43	BY4741, *trx2Δ::kanMX4 urm1Δ::KlURA3*	This study
FEY47	BY4741, *urm1Δ::kanMX4 trx1Δ::SpHIS5 trx2Δ::KlLEU2*	This study
RK101	BY4741, *urm1Δ::kanMX4 trr1Δ::SpHIS5*	This study
RK53	BY4741, *yap1Δ::kanMX4 urm1Δ::SpHIS5*	This study

Supplemental Table S2. Primers used in this study.

Primer	Sequence (5'-3')	Application
AHP1_C31S_FW	CAGTGAATCTAGTAAGATGCCACAAAC	*AHP1* SM *
AHP1_C31S_RV	GTTTGTGGCATCTTACTAGATTCACTG	*AHP1* SM
AHP1_C62S_FW	CTTTCTCCCCAACCAGTACTGTCAGCCATATTC	*AHP1* SM
AHP1_C62S_RV	GAATATGGCTGACAGTACTGGTTGGGGAGAAA	*AHP1* SM
AHP1_C120S_FW	CGACCCAGGCAGTGCTTTCACCAAATC	*AHP1* SM
AHP1_C120S_RV	GATTTGGTGAAAGCACTGCCTGGGTCG	*AHP1* SM
AHP1_F58A_FW	CCGGTGCTCCAGCTGCTGCTTCCCCAACCTGTACTG	*AHP1* SM
AHP1_F58A_RV	CAGTACAGGTTGGGGAAGCAGCAGCTGGAGCACCGG	*AHP1* SM
AHP1_F95A_FW	GTTACTGTTGACAACCCGGCTGCTAACCAAGCGTGGGC	*AHP1* SM
AHP1_F95A_RV	GCCCACGCTTGGTTAGCAGCCGGGTTGTCAACAGTAAC	*AHP1* SM
AHP1_K32R_FW	CAGTGAATCTTGTAGGATGCCACAAAC	*AHP1* SM
AHP1_K32R_RV	GTTTGTGGCATCCTACAAGATTCACTG	*AHP1* SM
AHP1_K156R_FW	CTTACGCTGCCAGGGAAACCAACC	*AHP1* SM
AHP1_K156R_RV	GGTTGGTTTCCCTGGCAGCGTAAG	*AHP1* SM
AHP1_FW_HindIII	GGGAAGCTTCCTTGGCCTCGATCTATTGC	Sequencing
AHP1_RV_EcoRI	GGGGAATTCCTGCTCCAACTCACTCTGTC	Sequencing
KO_URM1_FW	CAATACTGATTTCTGATACTAAAACGAGATAGGTTAATAGCA AAATCGGGCAGCTGAAGCTTCGTACGC	*URM1* KO **
KO_URM1_RV	CTTTATATATATATATGTAGCTGCTTCTTAAAAATTATTTGCT GCTATTTGCATAGGCCACTAGTGGATCTG	*URM1* KO
AHP1KOF	ATTTCAACAAACCAGAACAACACAAGTACTACCAATAACCAC AACAAAACCAGCTGAAGCTTCGTACGC	*AHP1* KO
AHP1KOR	TTTTGAATTTTTTTTATATAAACATGGTTTTATTGTCTATTACA TAGCATGCATAGGCCACTAGTGGATCTG	*AHP1* KO
KO_TRX1_FW	CCCTGAAACTGCATTAGTGTAATAGAAGACTAGACACCTCG ATACAAATACAGCTGAAGCTTCGTACGC	*TRX1* KO
KO_TRX1_RV	TATATAACAAACACAGTATAGAAACACAATATATCGGTCATT GGGTGAGTGCATAGGCCACTAGTGGATCTG	*TRX1* KO
KO_TRX2_FW	CACGCACACATACACGAGAGTCTACGATATCTTTAAATAACA CATCAATACAGCTGAAGCTTCGTACGC	*TRX2* KO
KO_TRX2_RV	TTTAAACTGGTAAACATGATGTACTTTACGTAGCGTTAATAT ACCGGCAAGCATAGGCCACTAGTGGATCTG	*TRX2* KO
KO_TRR1_FW	CTATACAGCAAATAGCGAACAGTACGAAAGTAAACATCATAT TATCAATACAGCTGAAGCTTCGTACGC	*TRR1* KO
KO_TRR1_RV	GATAAGTATACAAAAATTTGAGTGTATCTATTTTATAATGGAA AATTCATGCATAGGCCACTAGTGGATCTG	*TRR1* KO

* SM: site-directed mutagenesis ** KO: gene knock-out generation

Supplemental Table S3. Plasmids used in this study.

Plasmid	Description	Reference
pRS426	2µ ori, *ScURA3*	[81]
YCplac33	*ARS1-CEN4*, *ScURA3*	[80]
YCplac111	*ARS1-CEN4*, *ScLEU2*	[80]
pHA-URM1	*HA-URM1* cloned into pRS426$_{SmaI}$	[17]
pCB45	$_{P}$*GAL1-TAP-URM1* cloned into YCplac33$_{HindIII/SalI}$	[20]
pAJ46	$_{P}$*ADH1-(HA)3-hURM1-TCYC1* cloned into YCplac33$_{HindIII/SalI}$	[20]
pAJ31	*AHP1* cloned into YCplac111$_{HindIII/EcoRI}$	This study
pAJ33	*C31S* in pAJ31 by SM	This study
pAJ35	*K32R* in pAJ31 by SM	This study
pAJ39	*C62S* in pAJ31 by SM	This study
pAJ55	*K156R* in pAJ31 by SM	This study
pAJ57	*K32,156R* in pAJ31 by SM	This study
pAJ67	*C31,62S* in pAJ31 by SM	This study
pLK1	*C120S* in pAJ31 by SM	This study
pLK2	*C31,120S* in pAJ33 by SM	This study
pLK3	*C62,120S* in pAJ39 by SM	This study
pLK90	*C62S* in pAJ35 by SM	This study
pLK91	*C62S* in pAJ55 by SM	This study
pLK92	*C31S* in pAJ55 by SM	This study
pLK93	*C62S* in pAJ57 by SM	This study
pCiB40	*F58A* in pAJ31 by SM	This study
pCiB19	*F95A* in pAJ31 by SM	This study
pCiB39	*F58,95A* in pCiB19 by SM	This study
pMT173	ScUrm1-COSH in Intein system	[29]

3) Supplemental References

[17] K. Furukawa, N. Mizushima, T. Noda, and Y. Ohsumi, A protein conjugation system in yeast with homology to biosynthetic enzyme reaction of prokaryotes, J Biol Chem 275 (2000) 7462-7465.

[20] A. Jüdes, F. Ebert, C. Bar, K.L. Thuring, A. Harrer, R. Klassen, M. Helm, M.J. Stark, and R. Schaffrath, Urmylation and tRNA thiolation functions of ubiquitin-like Uba4.Urm1 systems are conserved from yeast to man, FEBS Lett 589 (2015) 904-909.

[29] M. Termathe and S.A. Leidel, The Uba4 domain interplay is mediated via a thioester that is critical for tRNA thiolation through Urm1 thiocarboxylation, Nucleic Acids Res 46 (2018) 5171-5181.

[80] R.D. Gietz and A. Sugino, New yeast-Escherichia coli shuttle vectors constructed with in vitro mutagenized yeast genes lacking six-base pair restriction sites, Gene 74 (1988) 527-534.

[81] T.W. Christianson, R.S. Sikorski, M. Dante, J.H. Shero, and P. Hieter, Multifunctional yeast high-copy-number shuttle vectors, Gene 110 (1992) 119-122.
